# Combining fractional polynomial model building with multiple imputation

**DOI:** 10.1002/sim.6553

**Published:** 2015-06-10

**Authors:** Tim P. Morris, Ian R. White, James R. Carpenter, Simon J. Stanworth, Patrick Royston

**Affiliations:** ^1^ Hub for Trials Methodology Research, MRC Clinical Trials Unit at UCLInstitute of Clinical Trials and MethodologyAviation House, 125 KingswayLondonWC2B 6NHU.K.; ^2^ Medical Statistics DepartmentLondon School of Hygiene & Tropical MedicineKeppel StLondonWC1E 7HTU.K.; ^3^ MRC Biostatistics UnitInstitute of Public HealthRobinson WayCambridgeCB2 0SRU.K.; ^4^ NHS Blood and TransplantJohn Radcliffe HospitalOxfordOX3 9BQU.K.

**Keywords:** fractional polynomials, multivariable fractional polynomials, multiple imputation, missing data

## Abstract

Multivariable fractional polynomial (MFP) models are commonly used in medical research. The datasets in which MFP models are applied often contain covariates with missing values. To handle the missing values, we describe methods for combining multiple imputation with MFP modelling, considering in turn three issues: first, how to impute so that the imputation model does not favour certain fractional polynomial (FP) models over others; second, how to estimate the FP exponents in multiply imputed data; and third, how to choose between models of differing complexity. Two imputation methods are outlined for different settings. For model selection, methods based on Wald‐type statistics and weighted likelihood‐ratio tests are proposed and evaluated in simulation studies. The Wald‐based method is very slightly better at estimating FP exponents. Type I error rates are very similar for both methods, although slightly less well controlled than analysis of complete records; however, there is potential for substantial gains in power over the analysis of complete records. We illustrate the two methods in a dataset from five trauma registries for which a prognostic model has previously been published, contrasting the selected models with that obtained by analysing the complete records only. © 2015 The Authors. Statistics in Medicine Published by John Wiley & Sons Ltd.

## 1 Introduction

In medical research, it is common to investigate the association between a continuous variable *x* and some outcome *y*. A default approach is to assume this association is linear. In scenarios where linearity is in doubt, researchers will sometimes categorise *x* 1, 2, forcing *x* to operate in step functions placed at (ultimately arbitrary) cut points 3, 4, which makes this a poor solution. Smoothing is thus central in medical statistics. Two popular and flexible approaches to allowing smooth nonlinear associations are splines 5 and fractional polynomials (FP) 6. FP models, and the methods used to build them, have the attraction of simplicity that has commended them to applied methodologists and explains their use in applied research. The current paper aims to describe how FP models can be applied in the presence of missing data and does not consider using splines with missing data, although we note that because both approaches have their place 7, such work would be useful.

The article originally introducing FP models acknowledged some shortcomings 8 but, according to Google Scholar, has been cited over 1000 times (accessed on 25 January 2015). While methods for developing FP methods are well established with fully observed data, many of the datasets to which FP models have been applied in the past have contained incomplete covariates 9, 10, 11, 12.

Multiple imputation (MI) is a general approach to handling missing data. Missing values are imputed *M* > 1 times by draws from the posterior predictive distribution of a model, returning *M* rectangular datasets. Each of these is analysed identically with the model that would have been used in the absence of missing data, and the resulting estimates are combined using rules developed by Rubin 13.

In principle, it should be possible to combine MI with FP methods. However, MI was developed assuming the analysis model of interest is fixed and known, while the testing required to build FP models would need to be used in imputed data, making it difficult to combine the two 6. Researchers are at present faced with a choice between using MI with an analysis model that assumes linearity 14, building FP models in complete records 15, or using an *ad hoc* combination of MI with FP models 9, 10, 11, 12.

The aim of this article is to propose and evaluate techniques for combining FPs with MI. We begin by describing FP models and how they are built (Section 2) and briefly outlining MI (Section 3). The issues that arise when combining the two are explained (Section 4) and some solutions introduced (Sections 5, 6 and 7). Two simulation studies evaluate these methods (Sections 6 and 7). Finally, the methods for building models are applied to the analysis of a dataset from five trauma centres for illustrative purposes (Section 8) 14.

## 2 Fractional polynomials

For a regression model involving a single continuous covariate *x*, a univariable FP model of dimension *D*, termed ‘FP*D*’, has *D* terms in *x* and linear predictor

(1) $$\beta_{0}+\overset{D}{\underset{d=1}{\sum }}\beta_{d}x^{p_{d}}.$$

This is the linear predictor for a regression model – including nonlinear models such as logistic regression and Cox proportional hazards models. Values of *p*_*d*_ are typically restricted to the set *S* where

**Figure 1:**
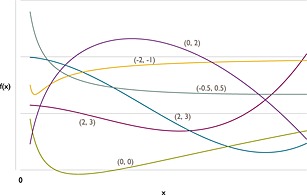
Example FP2 functions of the form $f(x)=\beta_{0}+\beta_{1}x^{p_{1}}+\beta_{2}x^{p_{2}}$. The numbers in parentheses are values of (*p*_1_,*p*_2_) used to plot the curve.

(2) S∈{−2,−1,−0.5,0,0.5,1,2,3},

which provides much practical flexibility. By convention, *x* ^0^= log*x*. Values of *x* must be strictly positive; for variables with negative values, 6 advises adding a constant to all values so that the smallest value is equal to the smallest increment between any two values. With *D* > 1, it is possible to have repeated powers for a covariate; the *d*‐th term is then taken as $x^{p_{1}}$, but the (*d* + 1)th is set to $x^{p_{1}}\log(x)$. For example, an FP2 logistic regression model with (*p*_1_,*p*_2_) = (−2,−2) would be

(3) $$\begin{array}{lll} \text{logit}(\pi_{i})=\beta_{0}+\beta_{1}x_{i}^{-2}+\beta_{2}x_{i}^{-2}\log x_{i}, & \; & \end{array}$$

where *π* is the probability that the binary outcome is 1. Values of *D* > 2 are rarely considered in practice, possibly because if such relationships are considered, plausible splines would be preferred. A variable thought to have a U‐shaped relationship with outcome would require *D* = 2. Meanwhile, *D* = 1 would be desirable for certain variables because it forces outcome to be a monotonic function of *x*_*c*_, and departures from this may be medically implausible. Figure 1 plots a selection of FP functions with *D* = 2, illustrating the range of curves on offer compared with linear functions, step functions or conventional polynomials.

The approach described in the preceding text can be extended to FP functions of multiple continuous covariates and is called a multivariable FP (MFP) model. With *C* continuous covariates *x*_1_,…,*x*_*C*_, the linear predictor is

(4) $$\beta_{0}+\overset{C}{\underset{c=1}{\sum }}\overset{D_{c}}{\underset{d=1}{\sum }}\beta_{\mathrm{cd}}x_{c}^{p_{\mathrm{cd}}}.$$

The *D*_*c*_ indicates that the complexity of the FP function may differ for different *c*.

### 2.1 Building fractional polynomial models

Methods for selecting FP models are described fully in 6 but summarised briefly here. There are two components involved in selecting models:

1. Estimation of *p*_*d*_ *for* *p*_*d*_=(*p*_1_,…,*p*_*D*_). This is performed by identifying the value of *p* that maximises the log‐likelihood and must be performed for each value of *d* considered in the next step.
2. Selection between models of different complexity. Likelihood ratios are used to test the simpler model (e.g. treating *x* as linear or omitting it altogether) against the best‐fitting FP*D*_max_model; if the test is significant at nominal level *α*, the next simplest model is tested versus the FP*D*_max_model, and so on. The selected model is the simplest for which the test against the FP*D*_max_model is not significant. If all tests are significant, the FP*D*_max_model is chosen.

In testing between models of different complexity, 1 degree of freedom (df) is assigned to each *β*, and 1 df to each *p*; thus, a test of FP1 versus FP2 is on 2 df. This assignment may lead to miscalibration of type I error rates for two reasons 16. First, ***β*** are estimated conditional on $\hat{p}$, treating $\hat{p}$ as fixed and known. The precision of confidence intervals around $\hat{\beta }$ is thus overstated. Second, the parameter space for $\hat{p}$ is discrete, constrained to taking values in *S*. The 1 df apportioned to each $\hat{p}$ estimated assumes the parameter space is continuous in (−*∞*,*∞*). This is not the case, meaning the 1 df is overly generous, implying conservatism in the testing procedures 16.

Building MFP models involves repeated application of the FP procedure to each *x*_*c*_ in turn 6. First, $x_{c^{\prime }}=(x_{1},\ldots ,x_{c-1},x_{c+1},\ldots ,x_{C})$ are treated as linear, and the FP selection procedure is applied to *x*_*c*_. The functional form of *x*_*c*_ is retained when FP is applied to *x*_*c* + 1_. This is applied to each variable in turn. The procedure is then run for the variables again conditioning on the current FP model, until the selected forms FPs are stable for a full cycle.

## 3 Missing data and multiple imputation

References 9, 10, 11, 12 all built FP models in partially observed datasets. The approaches used were *ad hoc*, so there is a need to understand and critique the potential approaches.

In a general context (not just FP models), MI is a flexible and popular approach to dealing with uncertainty due to missing data 17. Each missing value is imputed *M* > 1 times, producing *M* ‘complete’ imputed datasets. The analysis model that would be used for a complete dataset can then be fitted to each imputed dataset. The results of the *M* analyses are combined using rules described by Rubin 13, which can be used to combine estimators of population parameters.

By default, MI implementations assume data are ‘missing at random’ (MAR) or ‘missing completely at random’ (MCAR). These assumptions say that the probability of data being missing is independent of the missing values themselves; for MAR, this statement is conditional on the observed data. A more awkward assumption is ‘missing not at random’. MI implementations can be extended to missing not at random, but here we focus on MAR and MCAR.

Multivariate missing data can be imputed from a joint model, such as a multivariate normal or log‐linear model, or by ‘chained equations’ (often termed ‘fully conditional specification’, FCS or MICE) 17. The chained equations approach involves specifying a univariate imputation model for each incomplete variable conditional on other variables, and conditioning on current imputed values as covariates in the imputation of other variables. Incomplete variables are imputed in turn, and the process is repeated several (typically around 10) times.

If the models used for imputation and analysis are correctly specified, and under the assumption of MAR, MI provides an approximation to fitting a joint model for the distribution of covariates and outcome, leading to consistent estimates with nominal coverage. It is impossible to tell if the imputation and analysis models are correctly specified but it is desirable that the imputation model for incomplete covariates is at least ‘compatible’ with the analysis model, which is a necessary condition for the models to be correctly specified. Compatibility means that a joint model exists that implies both the imputation and analysis models as conditionals 18, 19.

A weaker condition is ‘semi‐compatibility’ 19, 20, 21, meaning the analysis model is compatible with a special case of the imputation model: the imputation model is ‘richer’ than the analysis model 17. These concepts are important in developing methods to combine FPs models with MI and are used in Section 5.

## 4 Difficulties in combining fractional polynomials with multiple imputation

Methods for building FP models with complete data are heavily reliant on likelihood‐ratio testing. In multiply imputed datasets, this approach is inappropriate, because we do not have a likelihood in MI data 17. With MI data, hypotheses would usually be tested using Wald statistics 22. However, the FP testing procedure does not obtain an estimate of $\mathrm{Var}(\hat{p})$ and so Wald statistics are not available. Procedures related to likelihood‐ratio tests do exist for MI data 22, 23 and are discussed in Section 7.

Imputation can become complex with FP models. Compatibility of the imputation and analysis models comes to the fore because when the analysis model is unknown, it becomes difficult to ensure that the imputation model is compatible or semi‐compatible.

The remainder of this paper aims to develop methods for dealing with these issues. First, we adapt two imputation methods to be used when the analysis involves FPs; second, we compare log‐likelihoods and Wald statistics to identify the ‘best‐fitting’ model of dimension *d*; third, we compare a testing procedure based on weighted likelihood ratios with one based on the model Wald statistic.

## 5 Imputing for fractional polynomials

Ahead of building an FP model, imputation must allow for the form of the FP functions that may be selected. If not, the imputation and analysis models may be incompatible, with consequences for estimation. Some options and our method of choice are outlined in the succeeding text.

### 5.1 Just another variable

Von Hippel developed an approach for imputing squared terms and interactions that ignores the true relationship between transformations and imputes functions as though the relationship was not deterministic, but estimated in the imputation 24. For example, *x* and *x* ^2^ may be treated as bivariate normal for the purposes of imputation. Seaman *et al*. later showed that the approach only works accurately for linear regression when *x* are MCAR and demonstrated potential for serious bias under departures from these conditions 25.

### 5.2 Predictive mean matching

Predictive mean matching has previously looked promising in settings where the imputation model is misspecified 17, either within von Hippel's approach 24 or by ‘passively’ imputing the nonlinear function from an imputed value of the original variable 25. However, although it can improve on parametric imputation assuming linearity, it has recently been shown to lead to bias in estimating nonlinear relationships 25, 26.

### 5.3 Substantive model compatible fully conditional specification

This imputation approach is based on rejection sampling. Bartlett *et al*. describe a method termed ‘substantive model compatible fully conditional specification’ (SMC FCS) and demonstrate that it can be used to impute squares and interactions in a way that is both compatible with the analysis model and respects the deterministic relationship between functions 21. Briefly, the method involves specifying a marginal distribution for *x*_*c*_, termed the proposal distribution, and rejecting or accepting proposal draws from this distribution with probabilities proportional to the likelihood of the observed outcome given the proposed value of *x*. This is embedded in a chained equations procedure where each *x*_*c*_ is imputed in turn.

SMC FCS is a general solution to imputation of nonlinear functions; Carpenter and Kenward give FPs as one example 27. However, they assume the FP functions to be included in the analysis model have been chosen at the point of imputation. To relax this assumption, one solution may be to allow for a very general form for *x*_*c*_ by including all candidate FP functions for the purposes of rejection sampling, which may be eight different transformations. This ensures the imputation models are semi‐compatible with whatever FP model is eventually selected.

The proposal is currently limited by two computational problems. The first is that imputed values of *x*_*c*_ must be positive so that FP transformations can be taken. Using a truncated model or predictive mean matching for drawing from the proposal distribution may resolve this. The second problem is collinearity. Even if the true model is truly a high‐dimensional FP, several of the variables may be collinear in the analysis model, leading to unstable rejection probabilities. If imputation was from a joint model, a suitable ridge parameter could be used to stabilise the model, but the method is based on chained equations, making the choice of an appropriate parameter difficult.

The rejection sampling method has potential but requires further thought to be usable for FP imputation problems, and it is not obvious how sensible dropping of collinear functions can be automated. One solution may be to specify a suitably flexible cubic spline model within the imputation step. In principle, this offers a similar flexibility to FPs. However, such an imputation model would imply that the final analysis model should also involve splines, and our aim is to develop imputation methods for FP analysis models. Further, a spline‐based imputation model is not compatible or semi‐compatible with an FP analysis model; the suggestion is based on both being flexible methods for modelling nonlinear effects. The usefulness of this approach would need verifying in simulations.

### 5.4 Drawing exponents via bootstrapping

The difficulty with imputation for FPs is in incorporating uncertainty about ***p*** in imputation models. When the posterior distribution is difficult to draw from, the approximate Bayesian bootstrap (ABB) can be a solution. A sample is drawn with replacement where individuals' probabilities of being resampled are drawn from a scaled multinomial distribution 28. For larger samples, this procedure becomes very similar inferentially to the bootstrap. We use the ABB to develop a method for imputing FP1 functions.

Consider an incomplete continuous covariate *x* with complete outcome *y*. The following imputation procedure is compatible with FP1 models for *y* on *x*:

1. Use ABB to draw a sample from the individuals with observed values of *x*.
2. For *p* =− 2(.)3, where (.)represents some small increment, fit a linear regression of *x* ^*p*^on *y* and any other covariates in the analysis model. This is compatible with the assumption that the analysis model is a regression model of *y* on *x* ^*p*^(and other covariates) for unknown *p*. Values in (.) must span the candidate powers considered by the analysis but could be less coarse. Increments of 0.2 are used in the present paper.
3. Find the value of *p* returning the largest value of log(*L*) + *J*, where *L* is the likelihood and *J* is the Jacobian for the transformation from *x* to *x* ^*p*^, required in order to make the log‐likelihoods comparable, and denote this value *p* ^*^. (As the maximum from a bootstrap sample, *p* ^*^is a nonparametric draw from the approximate posterior of *p*.)
4. Restore the partially observed dataset.
5. Impute missing ${\left(x\right)}^{p^{*}}$using linear regression of $x^{p^{*}}$on *y* and other variables from step 2.
6. Passively impute *x* ^*^by taking the *p* ^*^‐th root of ${\left(x^{*}\right)}^{p^{*}}$.

This procedure returns one of *M* imputed datasets.

As noted earlier, it is important that *x* ^*^ are positive, so that the standard FP transformations can be calculated for all *x* ^*^. We have implemented two options for imputation:

1. Impute using a truncated regression imputation model. Specify a (lower) truncation bound for *x* ^1^at some value >0and transform to a bound for $x^{p^{*}}$in step 5 (a lower bound for $p^{*}\geq 0$and an upper bound for *p* ^*^<0).
2. Perform the imputation in step 5 using predictive mean matching 17, 29, 30. Because the observed values of *x* are positive, the imputed values will be also.

### 5.5 Choice of imputation method

The current work uses the method based on the ABB for simulations, where the lower bounds are respected by drawing $x^{p^{*}}$ using predictive mean matching. However, the method described in the preceding text applies only to FP1 functions. For an extension to *D* = 2, a suitable approach may be to take the ‘polynomial combination’ approach of Vink and van Buuren to fit a model for all pairs of exponents (*p*_1_,*p*_2_) in an FP2 model 31. We note that such an extension would be extremely computationally intensive.

## 6 Estimation of exponents

The FP function selection procedure, which considers maximum dimension *D*_max_, requires estimation of the best‐fitting FP*d* models for *d* = 1,…,*D*_max_ as well as the linear and (possibly) null models. This section considers methods for estimating the best‐fitting FP*d* model in multiply imputed data.

### 6.1 Candidate methods

Wood, White and Royston consider methods for variable selection in multiply imputed data based on Wald tests and weighted likelihood‐ratio tests based on stacked MI data 22. We consider two related methods for the estimation of *p*:

1. Log‐likelihoods. The *M* imputed datasets are stacked and each FP*d* model fitted, treating the imputed datasets as a single complete dataset; $\hat{p}$is selected to maximise the log‐likelihood.
2. Wald statistics. $\hat{\beta }|p$and $\hat{\mathrm{Var}}(\hat{\beta }|p)$are estimated for all candidate *p* via Rubin's rules and the Wald statistic for testing *β* = 0calculated, with $\hat{p}$selected to maximise this quantity.

With complete data, the ‘best‐fitting’ FP*d* model is simply the one returning the largest value of the log‐likelihood. With multiply imputed datasets, the log‐likelihood is not meaningful for formal inferences, such as hypothesis tests. However, in comparing the fit of candidate FP*d* models with different values of *p*, the log‐likelihoods are not referred to any distribution. Because the models are of the same complexity, the ordering of competing FP*d* models by log‐likelihoods will be the same regardless of scale, so stacked observations need not be weighted.

Wald statistics have not previously been used for FP model building in complete data, and it is not clear whether they can be used to estimate *p*. However, Wald tests have previously been shown to be the ideal method for variable selection methods in MI data 22 and will be evaluated as the basis of testing procedures in Section 7; if they are also used to estimate *p*, then the overall procedure is more coherent.

If both of the aforementioned methods are unbiased, as expected, the method that estimates *p* with the greatest precision will be favoured.

### 6.2 Simulation design

To compare these methods, a simulation study based on FP1 is used. The true model involves linear regression of a continuous outcome *y* on an FP1 function of a continuous covariate *x*. Because we aim to compare bias and precision of log‐likelihoods with Wald statistics for estimating *p*, we use a larger set *S* here than the usual eight transformations given in (2). This does not impact on the methods themselves but provides a finer picture of bias and precision for the purpose of comparing methods.

The simulation procedure is as follows.

1. Complete data are simulated on *n* = 300observations from a bivariate normal distribution with parameters
  (5) $$(y,x^{p})\;∼\text{BVN}\left(\left[\begin{array}{c} 0\\ 3 \end{array}\right],\left[\begin{array}{cc} 1 & 0.7\\ 0.7 & 1 \end{array}\right]\right).$$
  This implies the true analysis model is a linear regression of *y* on *x* ^*p*^. It is important to produce a strong association between *x* and *y*, such that power for the true analysis model is close to 100% (i.e. if we fix $\hat{p}$ to equal *p*, then the test of *β* = 0 has almost 100% power). If Corr(*y*,*x* ^*p*^)≈0 in any simulated dataset, the profile for $\hat{p}$will be flat regardless of true *p*, and it becomes impossible to distinguish between good and bad methods with respect to estimation of *p*. In the context of prognostic models, where MFP models are particularly useful, *n* = 300may be regarded as a relatively small sample size 7.
2. Forty per cent of values of *x* are set to missing under a MAR mechanism such that the probability of *x* being missing is 0.2 when *y*≤0and 0.6 when *y* > 0.
3. Missing values in *x* are multiply imputed using the bootstrap method outlined in Section 5.4.
4. For *p*′=−2(.2)3, the linear regression analysis model for $\left(y|x^{p^{\prime }}\right)$is fitted and the log‐likelihood and Wald statistics based on MI data recorded. The log‐likelihood for complete data and complete records analyses is also recorded.
5. $\hat{p}$ is estimated as the value of *p*′maximising the log‐likelihood or Wald statistic.

This process is repeated a total of 10 000 times for true *p*= 0, 0.5, 1 and 2, and results are summarised graphically.

### 6.3 Simulation results

**Figure 2:**
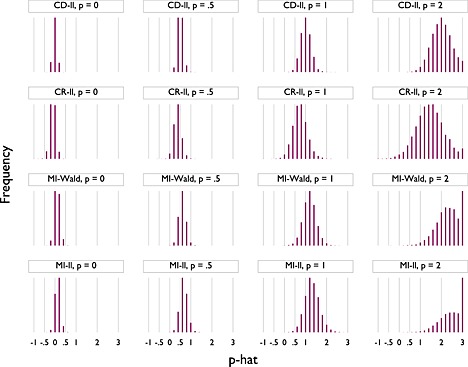
Simulation results: estimation of $\hat{p}$ according to method (10 000 replicates). CD‐ll is log‐likelihood in complete data; CR‐ll is log‐likelihood in complete records; MI‐Wald is Wald statistic in multiple imputation (MI) data; MI‐ll is log‐likelihood in MI data.

The simulation results are displayed as a spike plot in Figure 2. The columns represent different true values of *p*: from left to right, *p* = 0,0.5,1 and 2. Rows represent different methods for estimating *p*: from top to bottom, complete data using the log‐likelihood (CD‐ll), complete records using the log‐likelihood (CR‐ll), Wald statistics based on MI data (MI‐Wald) and log‐likelihoods based on MI data (MI‐ll). The horizontal axes represent different values of $\hat{p}$ and are labelled with the exponents typically used in *S*. The vertical axes display the frequency with which a given value was selected over the 10 000 replications. The vertical axes all originate at 0, but the maxima are scaled individually to make each sub‐plot as clear as possible.

As Figure 2 shows, across all methods, the sampling variance of $\hat{p}$ increases with the magnitude of *p*. This occurs because (for example) when *p* = 2 in truth, $\hat{p}=3$ is closer to the true model than $\hat{p}=2$ is when in truth *p* = 1. That is, a cubic is closer to a quadratic than a quadratic is to a straight line.

With complete data, use of log‐likelihoods is unbiased and efficient, as expected. Data are MAR, and so, there is some bias associated with complete‐case analysis, as well as lower precision.

The MI‐Wald method exhibits a slight upward bias for *p*. This bias is lowest for *p* = 0, increasing slightly for each larger value of *p*. The Wald method is also less precise than using complete‐data log‐likelihoods but slightly more precise than complete‐records log‐likelihoods.

The MI log‐likelihood method also exhibits a small upward bias, which is slightly greater than the bias in the MI‐Wald method. Again, precision is lower than for complete data and higher than for complete records.

Wald statistics and log‐likelihoods based on multiply imputed data both offer an improvement over analysis of the complete records. With imputed data, Wald statistics appear to do slightly better than log‐likelihoods in terms of both bias and precision. However, the differences are small, particularly in relation to the set of powers in *S* typically used in FP models. In this example, complete records was the worst method, although sometimes only slightly worse. It is worth noting that its performance will degrade further with multiple incomplete covariates.

Both the log‐likelihood and Wald methods will be carried forward to the methods evaluated in the following section, which focuses on hypothesis testing.

## 7 Methods for fractional polynomial model selection in multiply imputed data

The candidate methods we consider for selecting between FP models of different dimension are outlined in the following text. These methods represent a way for researchers to use the MFP model‐building algorithm in MI data.

### 7.1 Weighted likelihood‐ratio tests based on ‘stacked’ data

Wood, White and Royston 22 proposed new methods for hypothesis testing in multiply imputed data based on log‐likelihoods, which naturally extend to MFP models. The methods, designated ‘stacking’, involved treating the *M* imputed datasets as one dataset of *n* × *M* observations. The best stacking method explored in 22, designated ‘W3’, involves weighting all observations by *w*_*c*_=(1 − *f*_*c*_)/*M*, where *f*_*c*_ is the fraction of missing data for the *c*‐th covariate 22. Equal weights are assigned to all observations for each test, but the weight changes according to the covariate under scrutiny.

The use of the fraction of missing data for calculating weights is an attempt to weight each variable back to the correct amount of information: *f*_*c*_ attempts to approximate the fraction of missing information 32. When the approximation holds, stacking will work well. This would require a complete outcome, values to be MCAR and a covariate with missing values to be uncorrelated with other covariates. These are strong conditions that are extremely unlikely to be met in practice. When they are not, stacking will perform less well, but it is of interest to investigate how quickly it degrades under departures from these conditions.

### 7.2 Wald and ΔWald tests

Wald tests based on Rubin's rules have previously been demonstrated to be valid and powerful for variable selection in MI data 22.

For FP model selection, consider a Wald‐based procedure for a single covariate *x*. For use with FPs, the standard Wald statistic versus a null model for the parameters (*β*_1_…*β*_*D*_) can be calculated using Rubin's rules. However, if this test is significant, it is not possible to calculate a Wald statistic to test between non‐nested models, say FP1 versus linear (Section 2). It is instead proposed to use the difference between two models' Wald statistics; we term this method ‘ΔWald’. This is motivated by the fact that with fully observed data, the Wald statistic approximates the likelihood‐ratio test.

Note that there is no guarantee that a ΔWald statistic will be positive. This may not be a problem for testing because a negative Wald statistic is not significant at any level, but such behaviour in the left tail of the distribution might flag unusual behaviour in the right tail.

Model selection proceeds on the basis of Wald tests where possible and ΔWald otherwise. The *χ* ^2^ reference distributions and their dfs are the same as those used in the function selection procedure with complete data.

There is reason to suspect the dfs will be conservative. Consider the test of FP1 versus a null model. The Wald statistic is calculated from *β*_*c*1_ and tested using $\chi_{2}^{2}$ as the reference distribution. The df comes from the two extra parameters, *p*_*c*1_ and *β*_*c*1_, as compared with the null model, but the Wald statistic is actually calculated from *β*_*c*1_, conditional on ${\hat{p}}_{c1}$, a single parameter. Conversely, recall from Section 2.1 that $\hat{\mathrm{Var}}(\hat{\beta })$ will be underestimated because it is estimated conditional on ${\hat{p}}_{c1}$, assuming that this is the true *p*_*c*1_. This results in the Wald statistic for *β* being too large. The two errors may cancel out to some extent.

For the remainder of this article, Wald tests calculated against a genuine null model and those calculated from the difference in Wald statistics will both be referred to as ‘ΔWald’.

### 7.3 Other methods

We considered evaluating two other approaches to this problem. A brief description and justification of their omission is given in the succeeding text.

The first approach is Meng and Rubin's likelihood‐ratio test for multiply imputed data 23. This is derived from the asymptotic equivalence of Wald and likelihood‐ratio tests and was developed as a convenience tool to avoid calculation and inversion of *M* variance–covariance matrices in high‐dimensional datasets. By aiming to approximate a Wald test, it will perform at best as well as the associated Wald test. In unpublished work, P. R. has found the test to have extremely low type I error rates and thus hopelessly low power for building FP models. We do not therefore consider the approach further here.

The second approach is that of Robins and Wang 33. While their approach is strongly theoretically, there are several practical difficulties 34.

Robins and Wang take a different approach to imputation: imputed values are drawn conditional on the observed data and the observed‐data maximum likelihood estimate rather than first drawing parameters of the imputation model from the posterior 33. The imputer must save datasets containing the score function of the imputation model and the derivative of the score function with respect to the parameters of the imputation model. The analysis model is then applied to the *M* stacked imputed datasets assuming observations are independent. The analyst must save a dataset and matrix containing the estimating equations of the analysis model and the derivative of these equations with respect to the parameters of the analysis model. The approach provides consistent variance estimation when the imputation and analysis models are incompatible, although it is unimpressive with small sample sizes 34.

While Robins and Wang's method has been implemented in some simple cases involving monotone missingness 34, the demands are too great to attempt any application to problems involving FPs, where even ‘standard’ imputation and analysis models tend to be complex. Hughes, Sterne and Tilling show that the gains of Robins and Wang's method are typically modest and disappear with small sample sizes 34. It is assumed that the requirements of Robins and Wang's method would be too much to expect of researchers looking to apply FP models to incomplete datasets.

### 7.4 Simulation studies investigating proposed testing procedures

The simulation studies presented in sections 7.4.1 and 7.4.2 investigate the error rates of model selection by complete records, ΔWald and stacking for FP1 models, comparing these with analysis of the complete data as the gold standard. All scenarios involve a continuous outcome and two covariates, *x*_1_ and *x*_2_. The outcome *y* has a linear predictor based on $x_{2}^{1}$ and an FP1 transformation of *x*_1_.

#### 7.4.1 Simulation design

The following simulation setup is replicated 5000 times for each setting investigated. Two sample sizes are used for all settings: *n* = 200 and *n* = 500.

Covariates are simulated from the model

(6) $$(x_{1}^{-0.5},x_{2})∼\text{BVN}\left(\left[\begin{array}{c} \mu_{1}\\ \mu_{2} \end{array}\right],\left[\begin{array}{cc} \sigma_{1}^{2} & \sigma_{1}\sigma_{2}\\ \sigma_{1}\sigma_{2} & \sigma_{2}^{2} \end{array}\right]\right).$$

The parameters of this model are important as FP transformations will have more or less effect depending on the coefficient of variation for the variable being transformed. An FP1 transformation for a variable with mean 5 and variance 1 may allow for a degree of nonlinearity, in that fitting all FP1 models may give fairly different log‐likelihoods. If the mean is increased but the variance remains the same, FP transformations of the new variable will be closer to linear, in that the log‐likelihoods for the FP1 models will be closer. This is why the default behaviour of the FP commands for *Stata* (Stata. College Station, TX: StataCorp LP.) is to perform a preliminary scaling of *x*. The parameter values used here are *μ*_1_=0.6 and *σ*_1_=0.2, implying *x*_1_ has mean 3 and variance 1 (approximately), and *μ*_2_=3 and *σ*_2_=1. The value of *σ*_1_ *σ*_2_ is set to 0 or 0.5 for two different scenarios.

The outcome *y* is simulated from

(7) $$y_{i}∼\text{N}\left(\beta_{0}+\beta_{1}x_{1i}^{-0.5}+\beta_{2}x_{2i},\sigma_{y}^{2}\right).$$

The linear predictor includes an FP1 function of *x*_1_ and a linear function of *x*_2_. The same value of *p*_1_ was used in (6) and (7) so that the joint distribution for the complete data is $(x_{1}^{-0.5},x_{2},y)∼\text{MVN}$. For investigations of type I error, *β*_1_ is set to 0. For investigations of power, *β*_1_ is chosen such that, with complete data, the test for inclusion of *x*_1_ has 90% power. Note that this means *β*_1_ changes for different values of *σ*_1_ *σ*_2_ and *n*. The true value of *p*_1_ was chosen as −0.5 because this is relatively far from 1, meaning the test for FP1 versus a straight line has a good degree of power. When complete data analysis had 90% power for a test of FP1 versus null, the test of FP1 versus linear had approximately 80% power.

Values of *β*_2_ are chosen such that the likelihood‐ratio test for inclusion of *x*_2_ has 90% power with fully observed data.

For the results presented in 7.4.2, missingness occurs in *x*_1_, *x*_2_ or both, while *y* is complete. For each of these scenarios, two missing data mechanisms are invoked. Let *R*_*c*_ be a binary variable equal to 1 if *x*_*c*_ is observed and 0 if *x*_*c*_ is missing, and let *π* denote P(*R*_*c*_=1). Under MCAR, we set *π* = 0.7. Under MAR, we set logit(*π*_*i*_) = *ω*_0_+*ω*_1_ *y*_*i*_, with *ω*_0_ and *ω*_1_ chosen so that 70% of data are observed and comparison of *R*_1_ with *y* returns an area under the ROC curve of 0.65, making *π* and the degree of MAR comparable across simulation settings. Here, the sign of *ω*_1_ is always negative so that missing data are more likely at high values of *y*.

Missing *x*_1_ values are imputed using the bootstrap method described in Section 5.4 using *M* = 10 imputations, 10 cycles of chained equations (if both *x*_1_ and *x*_2_ are incomplete), with predictive mean matching with respect to the bounds.

The function selection procedure is run for complete data, complete records and MI data using stacking and ΔWald. The nominal size of tests used is *α* = 0.1 throughout, following Ambler and Royston 16. The most complex function considered is FP1. This is first tested against the null model and then against a model including *x*_1_ as linear. The quantity of interest is the rejection rate for each method. When *β*_1_=0, this should be as close to *α* as possible, indicating control of the type I error rates; when *β*_1_≠0, this should be as close to 1 as possible, maximising power.

The scenario expected to best suit stacking is *σ*_1_ *σ*_2_=0 with *x*_*c*_ MCAR, because here, *f*_*c*_ will approximate the fraction of missing information. MAR and *σ*_1_ *σ*_2_=0.5 will provide a sterner test for stacking. The test of FP1 against a null model is based on a true Wald statistic. The test of FP1 versus linear will provide a tougher test because it is based on ΔWald.

#### 7.4.2 Simulation results

**Figure 3:**
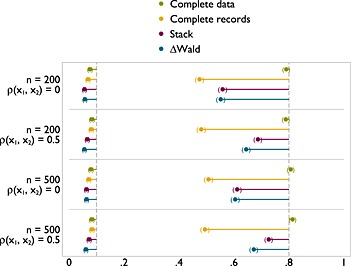
Type I error (left) and power (right) of FP1 versus linear test of nominal size 0.1 on *x*_1_ with *x*_1_ and *x*_2_ missing at random.

The results for MCAR and MAR were so similar as to be practically indistinguishable. Results are reported in the succeeding text for MAR only. Further, results were obtained for tests against a null model and tests against a linear model. The type I error rates were extremely similar for the two tests, and although power was (obviously) lower for the test of FP1 versus a linear model, the patterns over different methods are the same for all scenarios. The results for tests versus a linear model are shown here in Figure 3; the remainder of the results are given in Appendix but discussed here.

Figure 3 shows results for a scenario with both *x*_1_ and *x*_2_ are incomplete. The MFP model selection algorithm is run for both variables. Results are reported for tests relating to *x*_1_. The type I error for stacking and ΔWald is slightly further from 0.1 than analysis with complete data, or analysis of the complete records. However, this is very close, and at worst reaches 0.06. As might be anticipated, although power is never close to that of complete data, both stacking and ΔWald offer a substantial improvement over analysis of complete records. For the larger sample size, the gains in power are greater. Similar results are seen for tests versus a null model (Figure A.5).

**Figure A.2:**
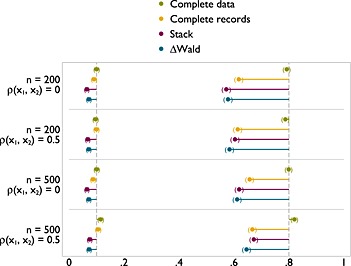
Type I error (left) and power (right) of FP1 versus linear test of nominal size 0.1 on *x*_1_ with *x*_1_ missing at random.

When *x*_1_ is incomplete but *x*_2_ is complete (Figures A.2 and A.1), complete data and complete records analyses have type I error rates very close to the nominal 0.1 level, while the type I error rates for stacking and ΔWald are slightly lower. When *x*_1_ is uncorrelated with *x*_2_, stacking has a slightly lower type I error rate than ΔWald; when there is correlation between *x*_1_ and *x*_2_, the two methods are more similar. Power for both stacking and ΔWald is relatively low in these scenarios, both being similar or slightly lower than complete records analysis in all scenarios. This implies that if the only incomplete covariate is the variable of substantive interest, complete records is as powerful as selecting a model in MI data.

**Figure A.4:**
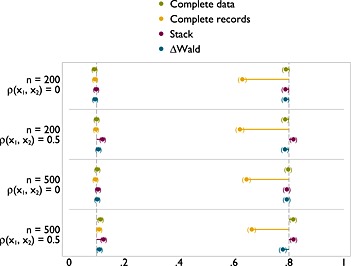
Type I error (left) and power (right) of FP1 versus linear test of nominal size 0.1 on *x*_1_ with *x*_2_ missing at random.

**Figure A.5:**
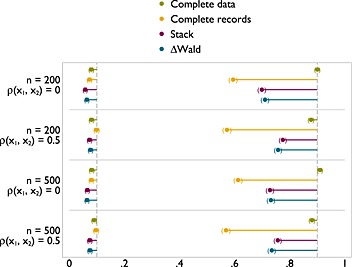
Type I error (left) and power (right) of FP1 versus null test of nominal size 0.1 on *x*_1_ with incomplete *x*_1_ and *x*_2_ missing at random.

When *x*_2_ is incomplete and *x*_1_ is fully observed, the type I error with respect to *x*_1_ is generally well controlled (Figures A.4 and A.3). It can be slightly high for stacking with *n* = 200 but not enough to cause concern. The type I error rate is well controlled by ΔWald throughout. Both methods can offer a substantial gains in power compared with complete records analysis. Meanwhile, power for ΔWald and stacking is extremely close to analysis of the complete data. Power is slightly higher for stacking than for ΔWald in settings where type I error is less well controlled. This demonstrates that power can be gained for *x*_1_ when *x*_2_ is incomplete, and so, using MI is appropriate.

#### 7.4.3 Conclusions on model selection

The aforementioned simulation studies demonstrate that both the stacking and ΔWald methods can be used to build MFP models in multiply imputed datasets.

The type I error is controlled to some extent by both methods. In our simulation studies, the type I error rates were 0.05 at the lowest and 0.14 at the highest for a test of nominal size 0.1. When a covariate of interest is incomplete but the outcome and confounder/s are complete, there may be little gain from using MI instead of complete records analysis: the type I error rates are lower, and power is very similar (although under MAR, complete records will lead to biased estimation of *p*; Section 6).

When a confounder is partially observed but the variable of interest is complete, the gains from using MI can be large. Type I error rates are higher than nominal in this setting but generally not enough to cause concern. The power gains of stack and ΔWald over complete records can be large here, coming close to the power of complete data analysis in the best scenarios (although when type I error rates differ, power is strictly not comparable).

When both the covariate of interest and a confounder are incomplete, results lie between the other two settings. Again, stacking and ΔWald have type I error rates that are too low – lower than complete data or complete records. Power can be gained for one variable when the other is subject to missingness.

The simulation study with both covariates incomplete is arguably closest to the way FP methods are most often used, which is for building prognostic models. In such settings, there will typically be several covariates with a complex missing data pattern. The results demonstrate that in such a setting, use of MI with stack or ΔWald will be beneficial, leading to an increased chance of correctly identifying the underlying relationships.

## 8 Prognostic model for massive transfusion: an illustration of building a fractional polynomial model in multiply imputed data

### 8.1 Data and published prognostic model

We illustrate the methods described and evaluated in the preceding text using a dataset of 5693 admissions to five trauma centres 14.

The publication associated with these data involved two main analyses. Our focus is on an analysis that developed a prognostic model for ‘massive transfusion’, defined as ≥10 red cell transfusions 14. The model was developed with the aim of facilitating appropriate, fast activation of major haemorrhage protocols by blood banks.

The development of a prognostic model was complicated by incomplete data on covariates. The variables measured and the frequency of missing values are given in Table 1. In total, 2456 (45%) of the 5693 individuals were complete records. Analysis of this subset would have potentially led to bias and the tests losing power for all variables.

**Table 1:** Summary of variables in the trauma dataset relevant to this work, *n*= 5,693.

	Frequency	Mean (SD)	Frequency (%)
Variable	missing (%)	in observed data	in observed data
Massive transfusion (outcome)	0 (0)		518 (9)
Age (years)	0 (0)	40 (20)	
Sex: male	0 (0)		4161 (73)
Injury type: penetrating	23 (0.4)		580 (10)
Time to emergency dept. (mins)	2396 (42)	65 (40)	
Systolic blood pressure (mm Hg)	425 (7)	126 (29)	
Base deficit (m*M*)	868 (16)	3.4 (5.1)	
Prothrombin time (seconds)	1,648 (29)	17 (8)	

SD, standard deviation.

In 14, data were assumed to be MAR. Multivariate imputation by chained equations was used to produce 50 imputations after 100 cycles. All variables in Table 1 were included in the imputation models. Injury type was the only incomplete categorical variable and was imputed using logistic regression. For continuous variables, transformations towards normality were taken before imputation, although for time to emergency department, this transformation was unsatisfactory, and so, predictive mean matching was used with a ‘donor pool’ of the three closest individuals 17, 26, 30. The normalised transformations used for imputation were also the form in which covariates were included in the prognostic model. These transformations ensured that each conditional imputation model was compatible with the analysis model, but as a consequence, the analysis model could not be an FP.

In 14, the prognostic model performed reasonably well. Validation in an external dataset produced an area under the ROC curve of 0.81, although predicted probabilities were often too low, demonstrating some miscalibration and/or differences between the training and validation data. The model was deemed not to be sufficiently accurate to use in practice, and so, the authors recommended against its adoption by emergency departments.

‘Missing at random’ implies that missing values did not depend on unobserved data. The assumption is questionable: if, for example, the probability of observing base deficit depends on prothrombin time, MAR would be false. Modelling the possible missing‐not‐at‐random mechanism is not the concern of the present paper, but this example analysis must be interpreted with this in mind.

### 8.2 MFP models with MI data

Without missing data, the analysis would have involved a logistic regression model with FP transformations for continuous predictors. This was not performed because it was not clear how to tackle the MFP algorithm with MI data.

In the following analyses, we use the multiply imputed datasets used in the published analysis. This means that the imputation may be incompatible with the final MFP model, although the purpose of the analysis is to demonstrate the two approaches to model building that were developed earlier. We compare the FP models selected using likelihood‐ratio tests in complete records with stacking and ΔWald in the MI data. The covariates included in the algorithm are sex (binary), age (continuous, *D*_*c*max_=2), time to emergency department (continuous, *D*_*c*max_=1), penetrating injury (binary), systolic blood pressure (continuous, *D*_*c*max_=2), prothrombin time (continuous, *D*_*c*max_=2) and base deficit (continuous, *D*_*c*max_=2).

Because the number of candidate predictors is relatively small, the FP analysis aims for caution with respect to omitting covariates completely by performing the test of FP*D*_*c*max_ versus null with nominal significance set at *α* = 0.5, meaning variables with little influence on the probability of massive transfusion can be excluded, but they will be included unless significance is extremely low. For the remaining tests, the significance level is set at *α* = 0.1.

### 8.3 Results of reanalysis

Table 2 shows the variables and exponents selected in complete records and in the MI data by stacking and ΔWald. For all three methods, convergence was achieved after two cycles through the FP selection algorithm.

**Table 2:** Models selected in trauma data.

	Complete records	Stack	ΔWald
Age (years)	−2	0.5, 1	1, 1
Sex[Link]	—	1	1
Injury type^*†*^ (blunt/penetrating)	1	1	1
Time to emergency dept. (minutes)	1	1	1
Systolic blood pressure (mm Hg)	1	1	−2, 0.5
Base deficit (m*M*)	1	−1	−0.5
Prothrombin time (seconds)	−0.5,−0.5	−0.5,−0.5	−0.5,−0.5

The numbers give the exponents selected for each variable in the final model.

† For binary variables, an exponent of 1 indicates inclusion in the final model.

Each method selected a different final model. Only time to emergency department, prothrombin time and injury type were included in the same form in all models. Complete records selected the simplest model overall, and ΔWald selected the most complex, although the model was similar to that selected by stacking.

The values of ${\hat{p}}_{c}$ selected by the models were sometimes different even when *D* was the same. For base deficit, *p*_*c*_=(−1) for stacking and (−0.5) for ΔWald. This is likely to be related to the result in Section 6, where stacking was shown to estimate ***p*** with slightly more bias than ΔWald. However, with multiple continuous variables subject to the model selection procedures, this can occur at any step of a cycle, and if the wrong form is selected for one variable, this can have a knock‐on effect on the form for the subsequent variables.

Because $\hat{\beta }$ are comparable conditional on $\hat{p}$ and *D*, comparing the values of $\hat{\beta }$ from the three selected models would be meaningless. Instead, the estimated FP functions are compared for age and base deficit from each of the three models for two notional individuals. The values used are invented but plausible representations of realistic individuals. The covariate values used are given in Table 3.

**Table 3:** Two notional individuals' covariate values used for Figure 4.

Individual	A	B
Age (years)	[Link]34	†24
Sex	Female	Male
Injury type	Blunt	Blunt
Time to emergency dept. (minutes)	63	73
Systolic blood pressure (mm Hg)	91	130
Base deficit (m*M*)	*13.5	*5.4
Prothrombin time (seconds)	16.8	14.4

**Figure 4:**
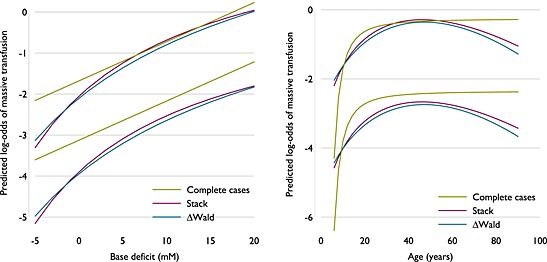
Fitted functions for two continuous variables (age and base deficit), by fixing parameters for other covariates, where the method of model selection returns different exponents.

* Values of age are fixed when base deficit is varied in Figure 4 and vice versa.

Figure 4 shows the comparison of fitted functions for these individuals across a range of values of age (from 6 to 90 years) and base deficit (from −5 to 20), both of which span most of the observed range of the covariates, while fixing other covariate values. Stacking and ΔWald return very similar fitted functions within the ranges considered, despite selecting slightly different $\hat{p}$. For both variables, the fitted functions for complete records are a completely different shape; in particular, the effect below age 10 years seems extreme.

## 9 Discussion

We have tackled the problem of combining MI with FP methodology, splitting the problem into three components: imputation and model building, which is split into estimation of exponents and selection of model complexity. The results of each component have been utilised and carried forward to the next component. Table A.1 gives a summary of the methods we have considered and our advice on their use in relation to FP models.

### 9.1 Imputation

Two approaches to imputation have been described. The first, based on the ABB, was used for the simulations of this paper. It has been developed to impute for FP1 functions but could in principle be extended. The rejection‐sampling approach is currently more general and should in principle work for larger values of *D*_max_. Neither method is controversial; both focus existing methods on the task of imputing for FP models. However, other approaches may exist that could improve on those suggested here.

In using rejection sampling or the ABB method to impute, both methods were noted as making imputation models semi‐compatible with the analysis model. For reasons of efficiency, it may be preferable to use a smaller imputation model and draw imputations from a model that is fully compatible rather than semi‐compatible with the analysis model. Consider FP models in complete data. Although $\hat{p}$ is ‘estimated’, it is subsequently treated as fixed and known. In the same spirit, it would be possible to impute initially, select the model and impute a second time, where the imputation model uses the selected FP functions. The selected model is then re‐fitted. This strategy may have advantages for the analysis: if the selected exponents are accurate, the restricted imputation strategies will result in ‘superefficient’ imputations 35. Conversely, if the exponents selected are inaccurate, the estimates after restricted imputation may compound errors. It is up to applied researchers to decide whether they are willing to take this risk in practice.

### 9.2 Model‐building algorithm

There are two distinct components to the algorithm used to build MFP models: estimating the best exponents for a covariate and selecting the appropriate complexity of FP function for that covariate.

The results of the simulations presented in Section 6 demonstrated that, for estimation of *p*, log‐likelihoods or Wald statistics from MI data are both superior to using log‐likelihoods based on complete records. This was with a single incomplete variable and one missingness mechanism; the performance of complete records could degrade further with other mechanisms and a more general pattern of missing data, although it would be unbiased under MCAR. Wald statistics appear to have lower bias than log‐likelihoods. Because the differences were only small, both methods were carried forward to model selection work, which assessed testing procedures based on stacking and ΔWald. It was judged to be advantageous to have a coherent method for estimation of ***p*** and variable selection: log‐likelihoods for stacking and Wald statistics for ΔWald testing.

These methods were evaluated in Section 7. Overall, the type I error rates for ΔWald and stacking were less well calibrated than for complete data or complete records; however, issues were not serious, and power could be higher even with lower type I error rates. The missing data mechanism and patterns in simulations were relatively simple, but complete records can become extremely inefficient with more complex missing data patterns, so whenever the proportion of complete records is low, it will be preferable to base the analysis on MI using stacking or ΔWald.

In practice, producing satisfactory imputations requires care. For building FP models in multiply imputed datasets, we advocate the use of ΔWald tests or stacking in preference to complete records analysis.

## Figures and Tables

**Figure A.1:**
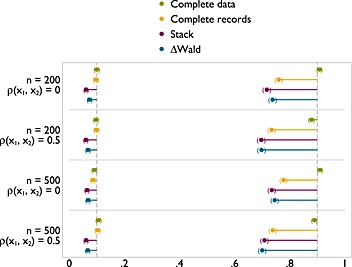
Type I error (left) and power (right) of FP1 versus null test of nominal size 0.1 on *x*_1_ with *x*_1_ missing at random.

**Figure A.3:**
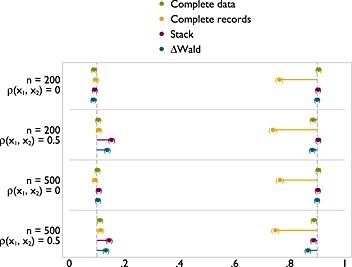
Type I error (left) and power (right) of FP1 versus null test of nominal size 0.1 on *x*_1_ with *x*_2_ missing at random.

## Appendix A

### A.1

A.1. Further results on rejection rates for model selection procedures

A.2. Summary of advice on methods for imputation and model selection

Several options for imputation, estimation of exponents and selection of model complexity are discussed in Sections 5, 6 and 7, respectively, and some evaluated and compared. Some options are dismissed and others recommended. Potential components of a strategy, and reasons for recommending or dismissing them in relation to combining FP models with MI, are summarised in Table  A.1 here.

A.3. Stata code fragments to implement recommended methods

This supplement gives examples of Stata code to implement some of our preferred procedures using a publicly available dataset in breast cancer, which can be downloaded and unzipped from http://portal.uni‐freiburg.de/imbi/Royston‐Sauerbrei‐book/index.html#datasets. The following analysis involves a logistic regression model relating recurrence or death (_d) to the number of positive lymph nodes, progesterone receptor status (fmol l^−1^) and age (in years).

The commands and help files for our recommended methods can be installed from the SSC (Boston College Statistical Software Components) repository. To download the packages, submit the following lines of code to Stata: . foreach pkg in smcfcs icet mfpmi mfpmi_wald mim {.     ssc install ‘pkg’. }The original dataset is complete. To introduce missing data in pgr and nodes, . replace pgr =. if runiform() < 0.2. replace nodes =. if runiform() < 0.2To multiply impute missing values 18 times using the method of 5.4 using predictive mean matching with 12 donors, . icet pgr nodes, add(18) method(pmm) knn(12) comp(age _d)/// powers(‐2(0.1)3)The following code selects a model using Wald tests to estimate *p* and select *D*_*c*_ for age, pgr and nodes. The df(#) option specifies the df allowed for the *D*_max_ model (2 indicates an FP1 and 4 indicates an FP2 model): . mfpmi_wald, df(nodes:2, pgr:4, age:4): logit _d age pgr nodesTo perform the same analysis using log‐likelihoods and stacking to select a model, the code is. mfpmi, df(nodes:2, pgr:4, age:4): logit _d age pgr nodesFinally, assume we have chosen the MFP model we wish to fit, which includes age ^−2^, age ^−2^ln(age), pgr ^.5^ and nodes ^.5^, as in 36. The following code creates the FP transformations before performing MI and fitting the analysis model using SMC FCS:

**Table A.1:** Possible strategies for imputation and model building with pros, cons and recommendations in light of results.

Stage	Possible approach	Pros	Cons	Practical advice
Imputation	JAV	Unbiased for linear models with data MCAR.	Biased in all other settings.	Avoid
	PMM	Ease of implementation; some ability to model nonlinear associations.	Performance degrades under strong MAR mechanisms.	Possibly useful for exploratory analysis.
	SMC FCS	Good approach if the analysis model is known, for example when validating a prognostic model.	Unclear how best to proceed when the analysis model is to be developed from multiply imputed data.	Consider using
	Draw FP1 exponents via ABB	Good approach if the highest dimension of FP considered is FP1.	Does not extend beyond FP1. Predictive mean matching can be incorporated for further flexibility but comes with the aforementioned caution. With complete binary or continuous covariates, the search over the parameter space of *p* becomes computationally infeasible.	Consider using
Estimation of *p*	Log‐likelihood in complete records	Reflects how MFP models are built in complete data. May be adequate with a small fraction of incomplete records and could be followed by SMC FCS to impute for the selected model.	Does not use incomplete records, leading to bias in estimates of $\hat{p}$ and $\hat{\beta }|\hat{p}$ under departures from MCAR.	Avoid unless there are few incomplete records
	Log‐likelihood in MI data	Reflects how MFP models are built in complete data but uses MI data.	Small bias in $\hat{p}$.	Consider using
	Wald statistics	Typically used in MI data where likelihoods do not have the same meaning.	Very small bias in $\hat{p}$ (less than using the log‐likelihood in MI data).	Consider using
Selection of *D*	Likelihood‐ratio tests on complete records	Type I error rate well controlled. May be adequate with a small fraction of incomplete records and could be followed by SMC FCS to impute for the selected model.	Estimates of *p* and *β* are biased. Power is lower than any alternative method.	Avoid unless there are few incomplete records
	Weighted likelihood‐ratio tests on stacked MI data 22	Standard approach to building MFP models in complete data. Superior power to complete records and less biased.	Approximation for the fraction of missing information may be wrong. Type I error rate less well controlled than analysis of complete records.	Consider
	Wald and ΔWald tests on MI data	The standard approach to testing in multiply imputed data. Better power and lower bias than complete records.	No theoretical basis for ΔWald. Type I error less well controlled than analysis of complete records.	Consider
	Meng and Rubin 23	Does not require access to full covariance matrix	Computational complexity and extremely low power.	Avoid
	Robins and Wang 33	Provides consistent variance estimation even when the imputation and analysis models are incompatible.	Impractical. Requires a different approach to imputation. Implementation is extremely complex for all but the simplest settings and is infeasible for MFP.	Avoid

ABB, approximate Bayesian bootstrap; JAV, just another variable; PMM, predictive mean matching; SMC FCS, substantive model compatible fully conditional specification; FP, fractional polynomial; MCAR, missing completely at random; MAR, missing at random; MFP, multivariable FP; MI, multiple imputation.
